# Fatigue Behavior of Rotary Friction Welding of Acrylonitrile Butadiene Styrene and Polycarbonate Dissimilar Materials

**DOI:** 10.3390/polym15163424

**Published:** 2023-08-16

**Authors:** Chil-Chyuan Kuo, Naruboyana Gurumurthy, Song-Hua Hunag

**Affiliations:** 1 Department of Mechanical Engineering, Ming Chi University of Technology, No. 84, Gungjuan Road, Taishan District, New Taipei City 24301, Taiwan; 2 Research Center for Intelligent Medical Devices, Ming Chi University of Technology, No. 84, Gungjuan Road, Taishan District, New Taipei City 24301, Taiwan; 3 Department of Mechanical Engineering, Chang Gung University, No. 259, Wenhua 1st Rd., Guishan Dist., Taoyuan City 33302, Taiwan; 4 Center for Reliability Engineering, Ming Chi University of Technology, No. 84, Gungjuan Road, Taishan District, New Taipei City 24301, Taiwan; 5 Department of Mechanical Engineering, Presidency University, Rajankunte, Near Yelhanka, Bangalore 700073, India; 6 Li-Yin Technology Co., Ltd., No. 37, Lane 151, Section 1, Zhongxing Road, Wugu District, New Taipei City 24101, Taiwan

**Keywords:** rotary friction welding, fatigue life, fatigue failure mechanism, number of cycles to rupture, rotational speed, cyclic load

## Abstract

Understanding the fatigue behaviors of weld joints is significant in engineering practice. Rotary friction welding (RFW) can join the additively manufactured polymer components. Until now, no research has focused on the fatigue behavior of polymer components jointed via RFW. This study investigates the fatigue life of ABS/PC dissimilar components fabricated via RFW and proposes the fatigue mechanism based on the failure structure. This work uses five different cyclic loads and rotational speeds to investigate the fatigue life. The fatigue life of the RFW of ABS/PC dissimilar rods is better compared with the pure ABS and pure PC specimens due to weld and integrity microstructural changes resulting from the combination of ABS and PC materials. The number of cycles until the rupture of RFW of ABS/PC dissimilar components (y) can be determined by the cyclic load (x) according to the prediction equation of y = −838.25x^2^ − 2035.8x + 67,262. The fatigue life of the RFW of ABS/PC dissimilar components increase with the increased rotational speed. The number of cycles until rupture (y) can be determined by the different rotational speeds (x) according to the prediction equation of y = 315.21x^2^ + 2710.4x + 32,124.

## 1. Introduction

The rotary friction welding (RFW) [1,2,3] of dissimilar materials is helpful in current industries, such as the automotive, aircraft, aerospace, and marine industries. RFW provides a lower energy consumption and environmental impact compared with fusion welding [4,5]. RFW needs very little heat and friction applied to the components during the welding process [6]. Therefore, this technology is frequently used to join metals or polymers [7]. Bhukya et al. [8] investigated its effect on the mechanical properties, downward force, and temperature profile of an aluminum alloy using friction stir welding. The results revealed that low force was decreased after introducing the copper donor. The surface hardness was reduced from the base metal to the center of the weld interface. Zhang et al. [9] predicted the fatigue life of the welding tool during the friction stir welding of an aluminum alloy. The results showed that the fatigue life of the welding tools was increased with the increase in the rotational speed. It was found that the compressive stress on the welding tool was at the back side and the tensile stress on the welding tool was at the front side. Shi et al. [10] employed laser welding to join the additive manufactured parts. The results indicated that the weld joint of the Al–Cu alloy was nearly free of defects. Liu et al. [11] reviewed the fatigue behavior of clinched joints, including their life estimation model, influencing factors, fatigue strength, and failure mechanism. Rayan et al. [12] investigated the fatigue behavior of maraging steel components. The results indicated that the mechanical property of maraging steel components was decreased by the reuse of maraging steel powder. Ahmed et al. [13] investigated the fatigue properties of aluminum alloys. The results showed that the weld joint with the 1.4 wt.% Mg filler provided the best fatigue life and highest fatigue strength. Su et al. [14] studied the fatigue behavior of tube connections under cyclic pressure. The results showed that the proposed method should be frequently used in un-welded and welded structures because it is easy to operate, is reliable, and is safe. Visco et al. [15] analyzed weld joints using static mechanical tests. The results revealed that the joints had an appreciable resistance to fatigue. Yu et al. [16] investigated the fatigue behavior of weld joints strengthened with carbon-fiber-reinforced polymer laminates. It was found that the predicted fatigue life was consistent with the experimental result. Koller et al. [17] identified an adhesive system suitable for achieving a high fatigue strength in a carbon-fiber-reinforced polymer patch. The results showed that the compressive nominal stress promoted the crack closure effect. Popescu et al. [18] investigated the fatigue behavior of polylactic acid orthoses. The results showed that the minimum force was approximately 95 N, reaching 110 N after 1100–1200 cycles during fatigue tests.

In the consumer electronics industry, acrylonitrile butadiene styrene (ABS) [19] and polycarbonate (PC) [20] are extensively employed in some critical components because they are more lightweight than metal. ABS has a high tensile strength and physical impacts. ABS plastic is suitable for making consumer products that withstand heavy use. PC plastic is also an engineering thermoplastic because it has excellent heat resistance. Therefore, both PC and ABS are widely used in the consumer electronics industry. However, few studies focus on the fatigue life of ABS/PC polymer rods jointed via RFW. According to practical experience, the welded parts’ reliability [21,22] is related to the fatigue life [23]. The main objective of this study was to investigate the fatigue life [24] of the polymer rods with five different cyclic loads. The fracture surfaces after the fatigue test were examined using an optical microscope (OM). Both fatigue life and fatigue behaviors were analyzed. Finally, the fatigue mechanism of the ABS/PC polymer rods was proposed.

## 2. Experimental Details

**Figure 1:**
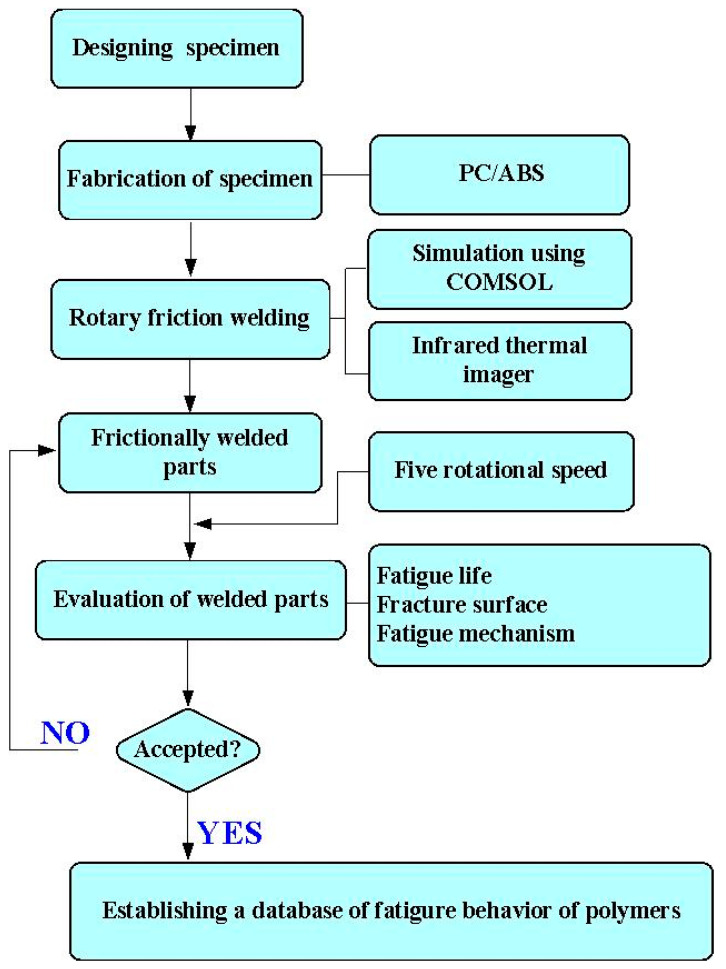
Flowchart of the research process used in this study.

**Figure 2:**
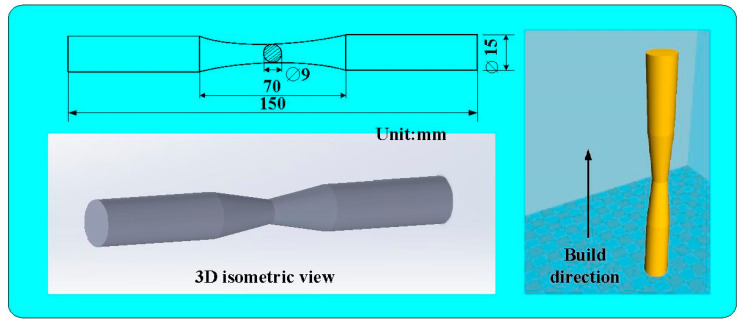
The size and geometry of the fatigue test specimen.

Figure 1 shows the flowchart of the research process used in this study. The objective was to study the fatigue behavior of the polymers. Figure 2 shows the size and geometry of the fatigue test specimen. The fatigue test specimen was also a cylindrical rod with a diameter of 15 mm and a length of 150 mm. The specimens were printed using a three-dimensional printing apparatus called fused deposition modeling (FDM) (Teklink smart solution Inc., New Taipei City, Taiwan) with two different kinds of thermoplastic filaments, i.e., ABS (Thunder 3D Inc., New Taipei City, Taiwan) and PC (Thunder 3D Inc., New Taipei City, Taiwan) [25,26,27]. The printing parameters for the ABS specimens involved a printing bed temperature of 100 °C, a printing speed of 80 mm/s, a printing temperature of 230 °C, and a layer thickness of 0.4 mm [28]. The printing parameters for manufacturing the PC specimens included a printing bed temperature of 100 °C, a printing speed of 80 mm/s, a printing temperature of 245 °C, and a layer thickness of 0.4 mm [29].

**Figure 3:**
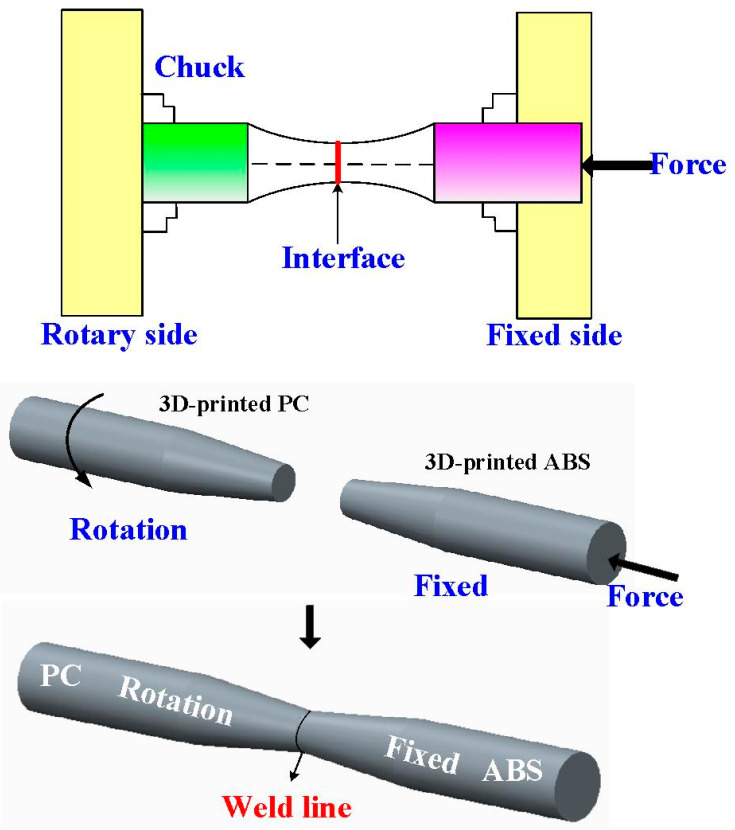
Schematic illustration of the RFW process used to make a fatigue test sample.

**Figure 4:**
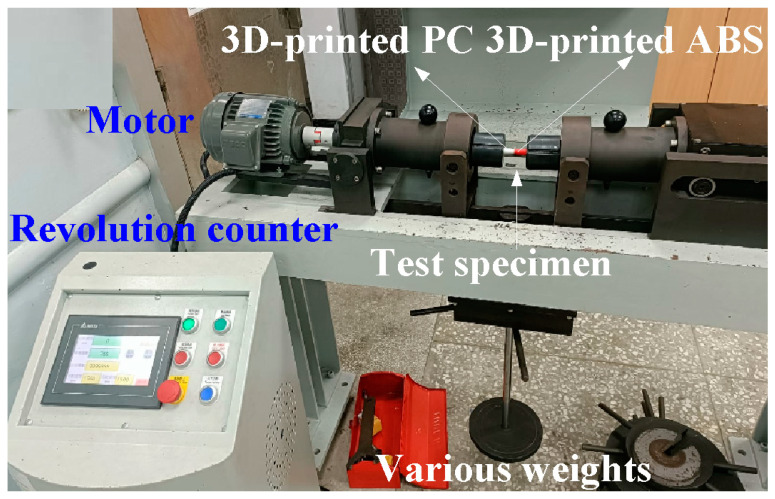
Experimental setup for the fatigue life of the welded parts.

In this work, a turning machine was used for RFW. The welding parameters included an axial load of 17 N, a feed rate of 0.1 mm/min, a friction time of 20 s, a welding time of 20 s, and a burn-off length of 2 mm. To investigate the effects of the rotational speed of RFW on the fatigue life of welded parts, five different rotational speeds were assessed in this study, i.e., 330, 490, 650, 950, and 1350 rpm. During RFW, the temperature history in the weld joint was recorded using an infrared thermal imager [30] (BI-TM-F01P, Panrico trading Inc., New Taipei City, Taiwan). Significantly, the temperature history in the weld joint was also predicted using COMSOL Multiphysics software. Figure 3 shows the schematic illustration of the RFW process used to make a fatigue test sample. After RFW, the fatigue tests were performed on pure ABS, pure PC, the RFW of ABS/ABS, the RFW of PC/ABS, as well as the RFW of ABS/PC rods using a rotating-beam fatigue test (3LMF03U801, Taiwan Nakazawa Co., Ltd., Taichung, Taiwan). Figure 4 shows the experimental setup for the fatigue life of the welded parts. To investigate the effects of loads on the fatigue life of the welded parts, five different loads were assessed in this work, i.e., 1, 2, 3, 4, and 5 kg. After the fatigue tests, the fatigue fracture surfaces were investigated comprehensively using an optical microscope. A fatigue mechanism was proposed according to the fatigue fracture surfaces. After the fatigue test, the fracture surface was analyzed using a stereo OM (Quick Vision 404, Mitutoyo Inc., Tokyo, Japan) and FE-SEM (JEC3000-FC, JEOL Inc., Tokyo, Japan).

## 3. Results and Discussion

**Figure 5:**
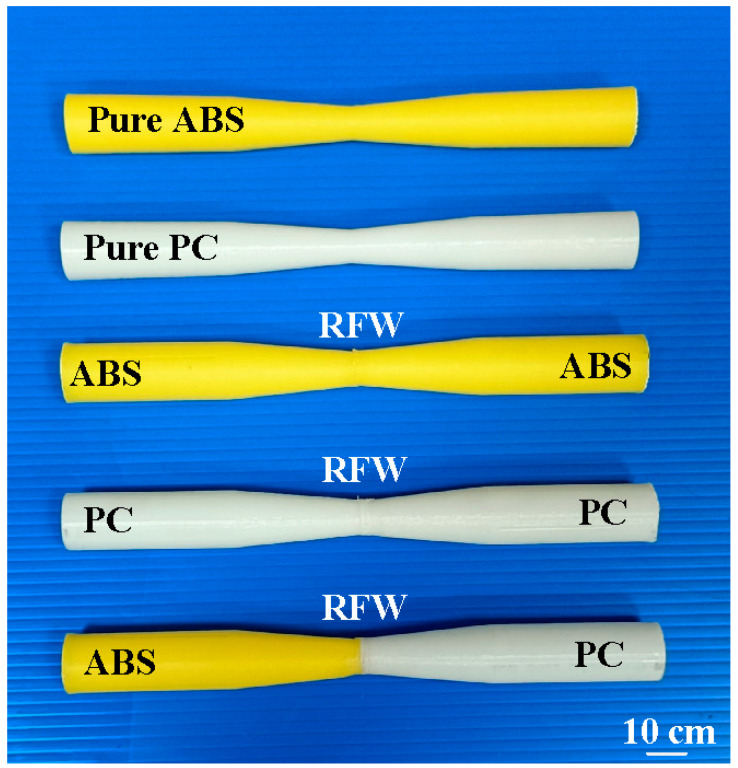
Test specimens for the fatigue test.

**Figure 6:**
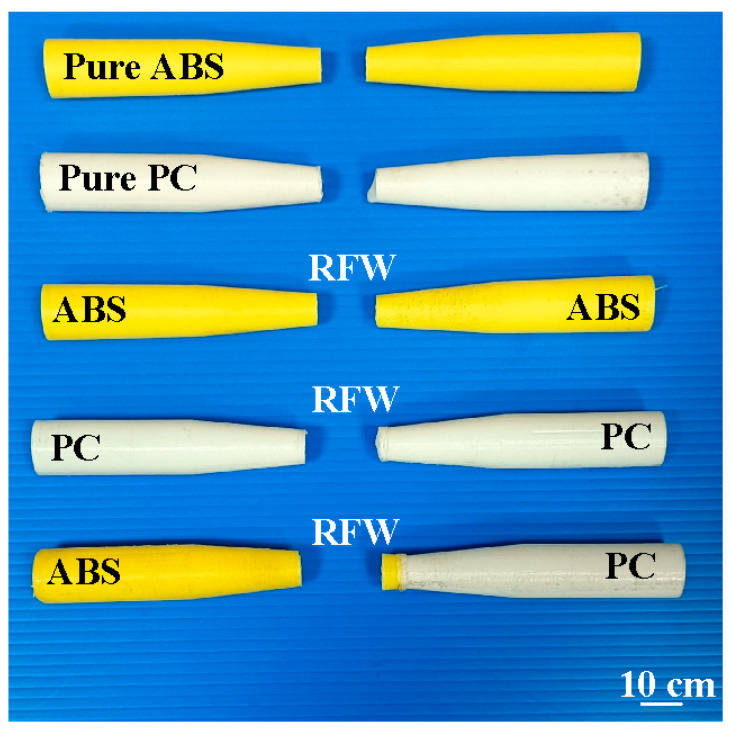
Fatigue test results.

**Figure 7:**
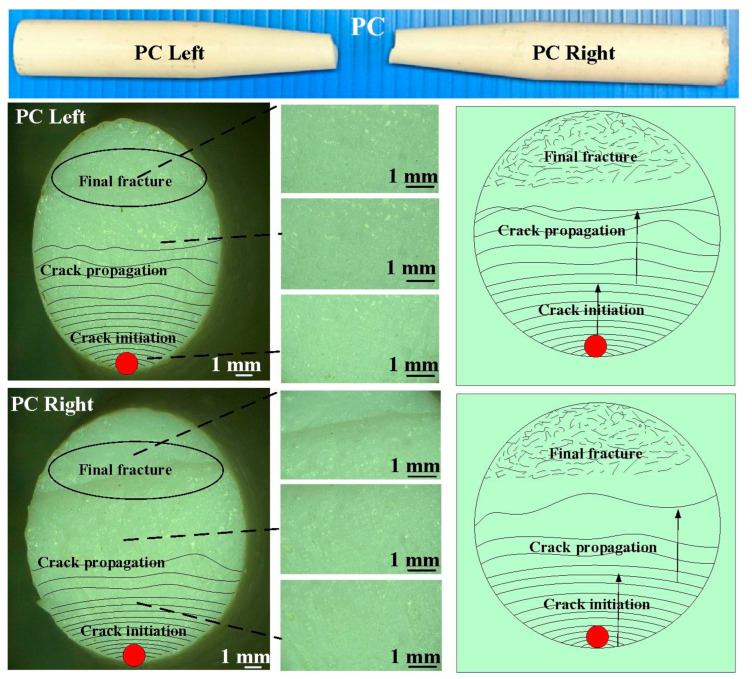
Fatigue failure surface of pure PC.

**Figure 8:**
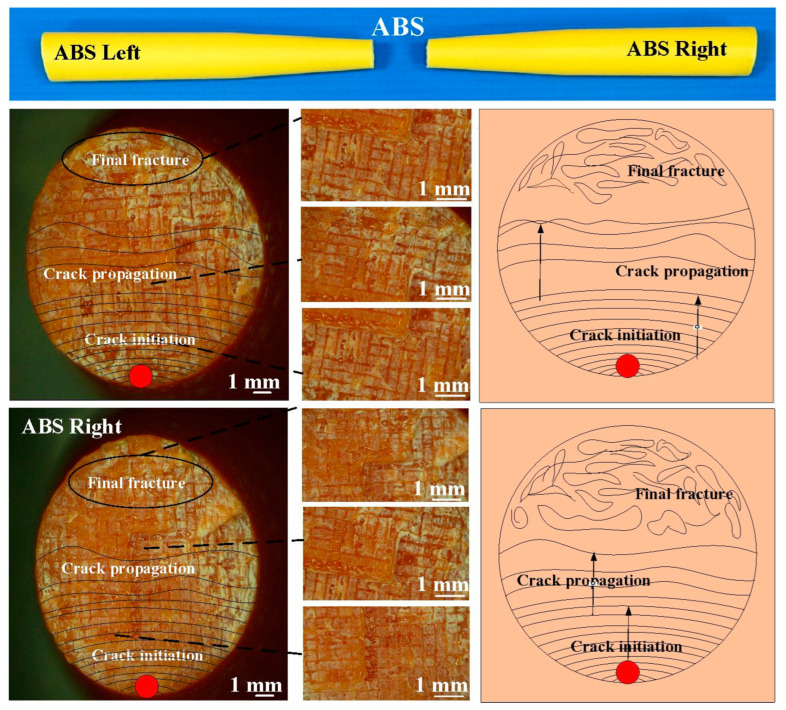
Fatigue failure surface of pure ABS.

**Figure 9:**
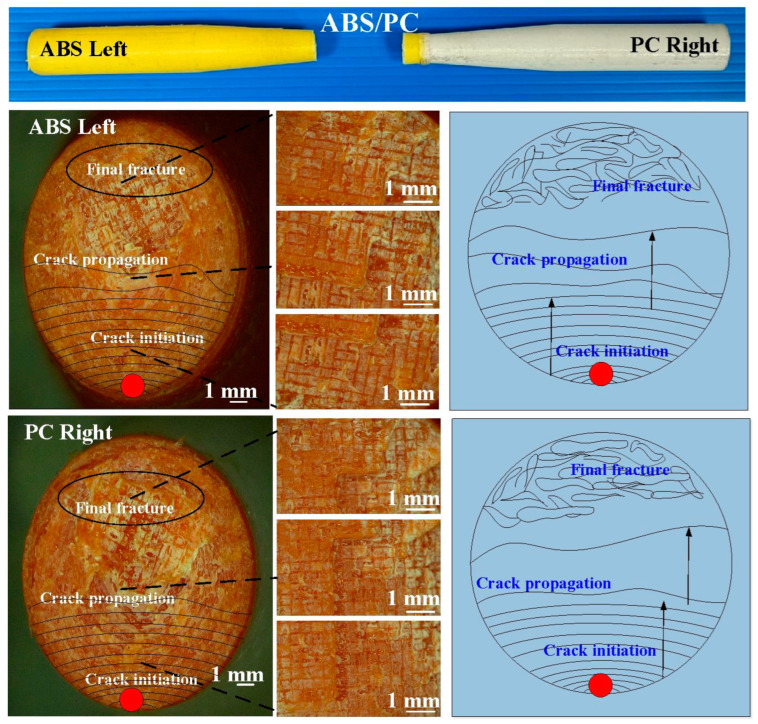
Fatigue failure surface of the RFW of ABS/PC.

The fatigue behavior of the RFW joints was evaluated using a rotating-beam fatigue-testing machine. In this study, three different fatigue samples were used to investigate the fatigue behavior. Two fatigue samples were 3D printed in PC and ABS using a fused deposition modeling apparatus. The other fatigue sample comprised 3D-printed PC and 3D-printed ABS joined using RFW. Figure 5 shows the test specimens for the fatigue test. After the fatigue tests, the fatigue fracture surfaces of solid ABS, solid PC, and the RFW of ABS/PC were investigated comprehensively using an optical microscope. Figure 6 shows the fatigue test results. For the welded part of the RFW of ABS/PC, the fractured location appears in the ABS polymer rod. For the welded part of the RFW of PC/PC, the fractured location appears in the weld interface. For the welded part of the RFW of ABS/ABS, the fractured location appears in the weld interface. Figure 7 shows the fatigue failure surface of pure PC. Figure 8 shows the fatigue failure surface of pure ABS. Figure 9 shows the fatigue failure surface of the RFW of ABS/PC. As can be seen, two distinct zones were found, i.e., a slow fracture zone and fast fracture zone. The original stands for the crack started during the fatigue test. In the fatigue zone, the crack grew gradually. The progression marks indicate the trend observed in the growth of the crack. In the overload zone, the crack grew quickly. Two different results were found. One is that the fatigue failure mechanism of polymers is the same as that of metals [31]. The other one is that the fatigue failure mechanism of welded parts produced via the RFW of PC and ABS dissimilar rods is the same as that of pure ABS or pure PC rods. This result shows that the welding quality of the RFW of PC and ABS dissimilar rods is robust. It should be noted that the fractured location appears in the ABS rods after the fatigue test, which is same as the bending test [32].

**Figure 10:**
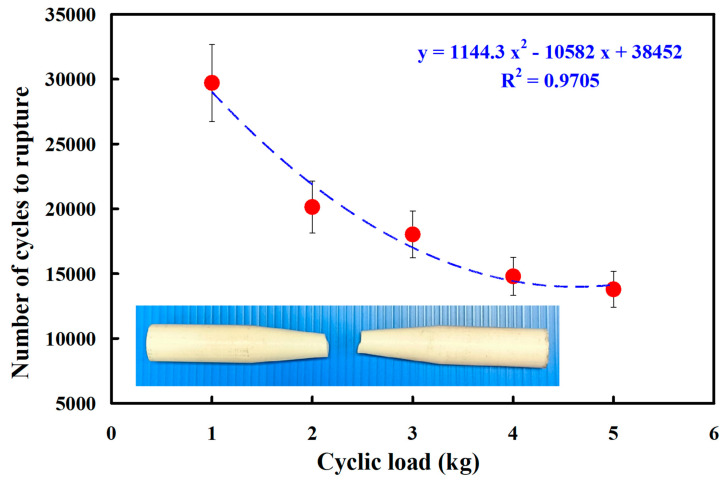
Fatigue test results of pure PC rods under five different cyclic loads.

**Figure 11:**
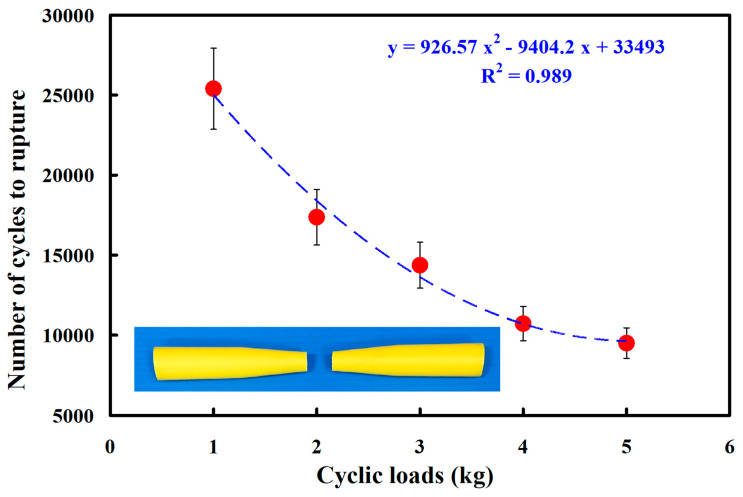
Fatigue test results of pure ABS rods under five different cyclic loads.

In this study, five specimens were assessed. Figure 10 shows the fatigue test results of the pure PC rods under five different cyclic loads. The results showed that the average number of cycles until rupture was about 29,706, 20,140, 18,024, 14,796, and 13,796 when the pure PC fatigue test specimens were subjected to five different loads of 1, 2, 3, 4, and 5 kg. As can be seen, the fatigue life of the pure PC fatigue test piece was shorter under a higher load. Based on the experimental results obtained for a cyclic load of 1 kg, the reduction ratios of the cycles until rupture for four different loads of 2, 3, 4, and 5 kg were about 32.20, 39.33, 50.19, and 53.56%, respectively. It should be pointed out that the average number of cycles until rupture (y) can be determined by the cyclic load (x) according to the prediction equation of 1144.3x^2^ − 10,582x + 38,452, with a correlation coefficient (R^2^) of 0.9705. Figure 11 shows the fatigue test results of the pure ABS rods under five different loads. The results showed that the average number of cycles until rupture was about 25,400, 17,370, 14,372, 10,718, and 9502 when the pure ABS fatigue test specimens were subjected to five different loads of 1, 2, 3, 4, and 5 kg.

As a result, the average number of cycles until rupture (y) can be determined by the cyclic load (x) according to the prediction equation of y = 926.57x^2^ − 9404.2x + 33,493, with a correlation coefficient of 0.989.

**Figure 12:**
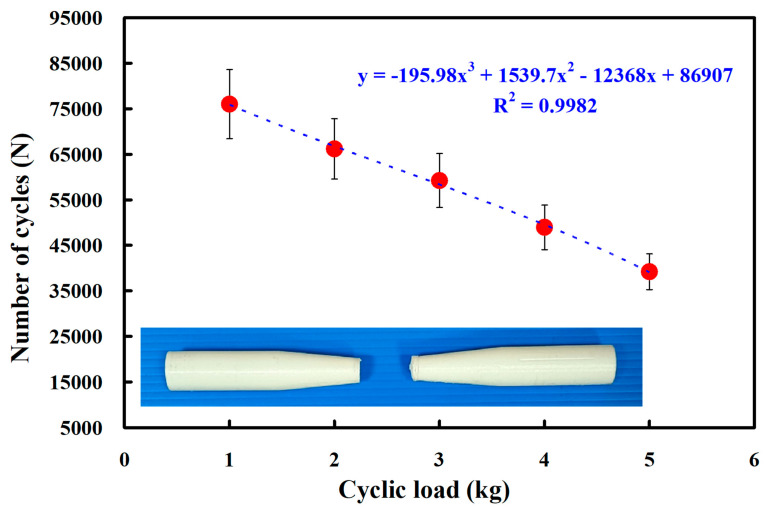
Fatigue test results of the RFW of PC and PC similar rods under five different cyclic loads.

**Figure 13:**
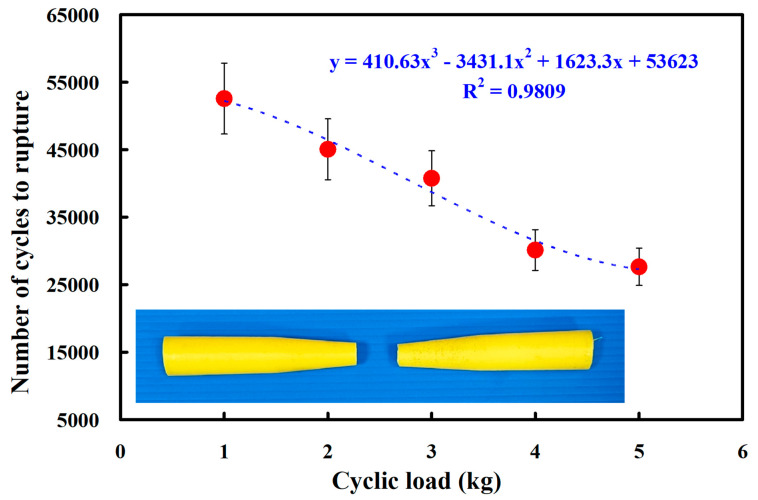
Fatigue test results of the RFW of ABS and ABS similar rods under five different cyclic loads.

Figure 12 shows the fatigue test results of the RFW of PC and PC similar rods under five different cyclic loads. The results showed that the average number of cycles until rupture was about 76,027, 66,185, 59,234, 48,950, and 39,205 when the fatigue test specimens were subjected to five different loads of 1 kg, 2 kg, 3 kg, 4 kg, and 5 kg. As a result, the number of cycles until rupture (y) can be determined by the cyclic load (x) according to the prediction equation of −195.98x^3^ + 1539.7x^2^ − 12,368x + 86,907, with a correlation coefficient of 0.9982. Figure 13 shows the fatigue test results of the RFW of ABS and ABS similar rods under five different cyclic loads. The results showed that the average number of cycles until rupture was about 52,569, 45,059, 40,758, 30,127, and 27,634 when the fatigue test specimens were subjected to five different loads of 1 kg, 2 kg, 3 kg, 4 kg, and 5 kg. As a result, the number of cycles until rupture (y) can be determined by the cyclic load (x) according to the prediction equation of 410.63x^3^ − 3431.1x^2^ + 1623.3x + 53,623, with a correlation coefficient of 0.9809.

**Figure 14:**
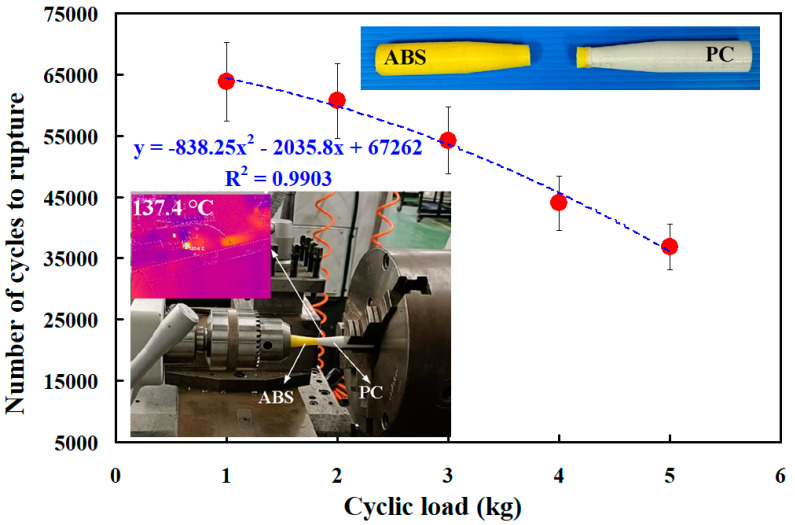
Fatigue test results of the RFW of ABS/PC dissimilar rods under five different cyclic loads.

**Figure 15:**
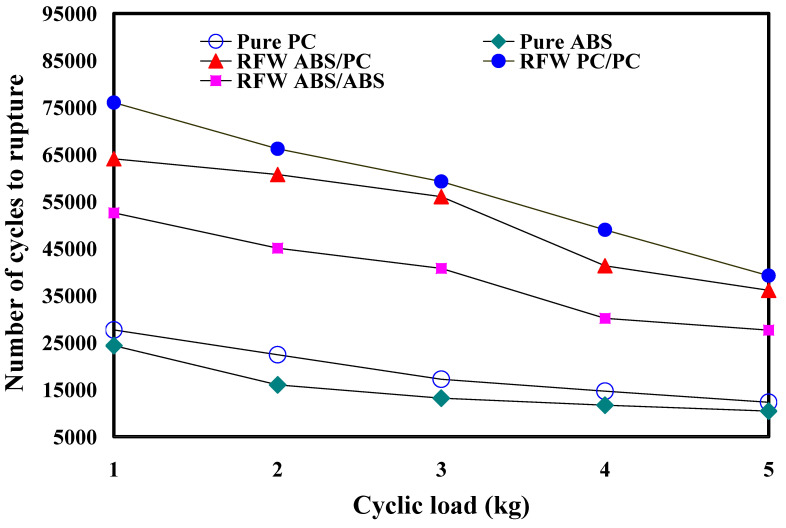
Fatigue test results of five different fatigue test specimens under five different cyclic loads.

Figure 14 shows the fatigue test results of the RFW of ABS/PC dissimilar rods under five different cyclic loads. The results showed that the average number of cycles until rupture was about 63,850, 60,701, 54,254, 43,988, and 36,880 when the fatigue test specimens were subjected to five different loads of 1, 2, 3, 4, and 5 kg. The number of cycles until rupture (y) can be determined by the cyclic load (x) according to the prediction equation of −838.25x^2^ − 2035.8x + 67,262, with a correlation coefficient of 0.9903. Figure 15 shows the fatigue test results of the five different fatigue test specimens under five different cyclic loads. It should be noted that the fatigue life of the RFW of PC/PC dissimilar rods is the best due to the weld integrity resulting from the combination of PC and PC materials after RFW [33]. The fatigue life of the RFW of ABS/ABS similar rods is the worst. The fatigue life of the RFW of ABS/PC dissimilar rods is in between. These results are consistent with the mechanical properties of the welding base metal [34,35,36].

**Figure 16:**
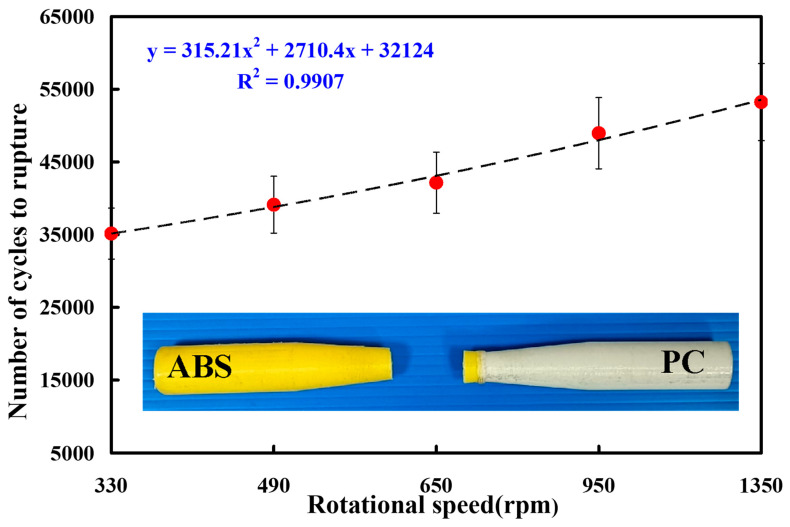
Fatigue test results of the RFW of ABS and PC dissimilar rods under five different rotational speeds.

Figure 16 shows the fatigue test results of the RFW of ABS and PC dissimilar rods under five different rotational speeds. The results showed that the average number of cycles until rupture of the RFW of ABS and PC using a rotational speed of 330 rpm, 490 rpm, 650 rpm, 950 rpm, and 1350 rpm was about 35,150, 39,120, 42,145, 48,957, and 53,240, respectively. It was found that the fatigue life of the RFW of ABS and PC increased with the increase in the rotational speed. This result shows that the weld strength in the weld interface was enhanced by increasing the rotational speed [37] due to the high peak temperature in the weld interface, resulting in high material flow during RFW. The average number of cycles until rupture (y) can be determined by the rotational speed (x) according to the prediction equation of 315.21x^2^ + 2710.4x + 32,124, with a correlation coefficient of 0.9907. It is interesting to note that the fatigue life of the RFW of ABS/PC is better than both the pure ABS and pure PC materials.

In general, RFW is a green manufacturing process that can reduce energy consumption compared with conventional arc welding. As a result, RFW meets the sustainable development goal 12 [38]. RFW is a practical method in various industries [39,40,41]. However, a lathe was used for the RFW in the current work. To reduce experimental error, the computer numerical control turning machine [42,43,44,45] was recommended to perform the RFW because the feed rate of RFW can be controlled precisely. In addition, the rotational speed [46] can be changed during the process of RFW. In addition, the rate of growth of a fatigue crack was not investigated using the Paris law [47,48,49]. Carbon dioxide laser [50,51,52] or fiber laser [52,53,54,55] have also been recommended as methods with which to join polymer rods. These topics are interesting research topics and are currently being investigated.

## 4. Conclusions

The main aim of this study was to investigate the fatigue life of polymer parts fabricated using RFW. Five different cyclic loads were used to study the effects of loads on the fatigue life. The main conclusions from the experimental work in this study are as follows:

1. The fatigue failure mechanism of polymers is the same as that of metals.
2. The fatigue life of the RFW of PC/PC dissimilar rods is the best due to weld and integrity microstructural changes resulting from the combination of PC and PC materials. This result shows that the welding quality of the RFW of PC/PC dissimilar rods is robust for application in various industries.
3. The fatigue life of the RFW of ABS/PC is shorter under higher loads. The number of cycles until failure (y) can be determined by the cyclic load (x) according to the prediction equation of y = −838.25x^2^ − 2035.8x + 67,262, with a correlation coefficient of 0.9903.
4. The fatigue life of the RFW of ABS/PC dissimilar components increases with an increased rotational speed. The number of cycles until rupture (y) can be determined by the rotational speed (x) according to the prediction equation of 315.21x^2^ + 2710.4x + 32,124, with a correlation coefficient of 0.9907.

## Data Availability

Data and materials are available.
